# Patients Receiving Integrative Medicine Effectiveness Registry (PRIMIER) of the BraveNet practice-based research network: study protocol

**DOI:** 10.1186/s12906-016-1025-0

**Published:** 2016-02-04

**Authors:** Jeffery A. Dusek, Donald I. Abrams, Rhonda Roberts, Kristen H. Griffin, Desiree Trebesch, Rowena J. Dolor, Ruth Q. Wolever, M. Diane McKee, Benjamin Kligler

**Affiliations:** 1 Integrative Health Research Center, Penny George Institute for Health and Healing, Allina Health, 2925 Chicago Avenue South, Mail Route 10039, Minneapolis, MN 55407-1321 USA; 2 Osher Center for Integrative Medicine, University of California San Francisco, San Francisco, CA USA; 3 Duke Clinical Research Institute, Duke University Medical Center, Durham, NC USA; 4 Duke Integrative Medicine, Duke University Medical Center, Durham, NC USA; 5 Department of Physical Medicine & Rehabilitation, Osher Center for Integrative Medicine, Vanderbilt Schools of Medicine and Nursing, Nashville, TN USA; 6 Albert Einstein College of Medicine of Yeshiva University, New York, USA; 7 Department of Integrative Medicine, Mount Sinai Beth Israel Medical Center, New York, NY USA

**Keywords:** Integrative Medicine, Observational study, Practice-based research, Depression, Stress, Patient activation, Complementary medicine, Study protocol

## Abstract

**Background:**

Integrative medicine (IM) provides patient-centered care and addresses the full range of physical, emotional, mental, social, spiritual, and environmental influences that affect a person’s health. IM is a “whole systems” approach that employs multiple modalities as opposed to an isolated complementary therapy. Thus, studying outcomes of IM is more challenging than evaluating an isolated intervention. Practice-based research networks (PBRNs) allow for clinicians/investigators at multiple diverse sites using common methodology to pool their data, increase participant sample size and increase generalizability of results. To conduct real-world, practice-based research, the Bravewell Collaborative founded BraveNet in 2007 as the first national integrative medicine PBRN.

**Methods and design:**

Patients Receiving Integrative Medicine Effectiveness Registry (PRIMIER) is a prospective, non-randomized, observational evaluation conducted at fourteen clinical sites. Participants receive a non-standardized, personalized, multimodal IM approach for various medical conditions. Using the REDCap electronic platform, an anticipated 10,000 study participants will complete patient-reported outcome measures including Patient Reported Outcomes Measurement Information System (PROMIS)-29, Perceived Stress Scale-4, and the Patient Activation Measure at baseline, 2, 4, 6, 12, 18 and 24 months. Extractions from participants’ electronic health records include IM services received, as well as ICD diagnostic codes, and CPT billing codes associated with each IM visit. Repeated-measures analyses will be performed on data to assess change from baseline through 24 months with planned subgroup analyses to include specific clinical population and specific IM intervention or combinations.

**Discussion:**

As the PRIMIER registry grows, we anticipate that our results would provide an indication of the promise of PBRN research efforts in IM. Analyses will incorporate a large sample of participants and an expected 10-year observation period and will provide the ability to evaluate the effect of IM on outcomes for specific clinical populations and specific IM interventions or combinations. As such, PRIMIER will serve as a national platform for future evaluations of IM best practices.

**Trial registration:**

Clinical Trials.gov NCT01754038

**Electronic supplementary material:**

The online version of this article (doi:10.1186/s12906-016-1025-0) contains supplementary material, which is available to authorized users.

## Background

Integrative medicine (IM) provides patient-centered care and addresses the full range of physical, emotional, mental, social, spiritual, and environmental influences that affect a person’s health [1]. Employing a personalized strategy that considers the patient’s unique conditions, needs, and circumstances, IM uses the most appropriate interventions from an array of scientific disciplines to heal illness and help people regain and maintain optimal health [1]. Because IM is a “whole systems” approach that employs multiple modalities in concert as opposed to an isolated complementary therapy, studying outcomes is more challenging than evaluating an isolated pharmaceutical or botanical intervention [2].

“Patient-reported outcome” (PRO) measures are health data provided directly by patients, rather than outcomes that reflect the assessment of an investigator or clinician, as typically seen in clinical research. A PRO is an individual patient’s self-reported assessment of their feelings or functions as they are dealing with diseases or conditions, and this assessment has important implications both in clinical practice and in research. PRO data are especially important in evaluating the effectiveness of health care for chronic conditions, where a primary goal of treatment is to improve patients’ function and to reduce symptoms associated with the condition [3, 4]. Criticisms of the use of PROs in clinical research have been the wide range of instruments used, often to measure similar outcomes, and the lack of standardization between outcome measures [4]. The recent development [5] of the NIH-funded Patient-Reported Outcomes Measurement Information System (PROMIS) suite of item banks and short forms for assessing health outcome domains related to quality of life and chronic conditions has been cited by researchers as a viable solution to these concerns [4, 6].

Single-site studies in IM are generally too small to identify differences in outcomes across sociodemographic or clinical subgroups. Multi-institutional scientific networks are able to recruit large numbers of participants for observational and interventional trials. Practice-based research networks (PBRNs) allow for clinicians/investigators at multiple diverse sites using common methodology and a single coordinating/data analysis center to pool their data as well as increase generalizability of results. By collecting data in the context of patients receiving their clinical care, the results are more reflective of “real world” outcomes. Given the growth of the number of clinics providing IM [7] and the resulting need for these clinics to conduct real-world, practice-based research, the Bravewell Collaborative founded BraveNet in 2007 as the first national IM PBRN. BraveNet, a registered PBRN with the Agency for Healthcare Research and Quality (AHRQ), was established with the intent that it would provide data on IM’s use, effectiveness, safety, costs, and patient satisfaction. As of early 2015, BraveNet consists of 14 IM clinics that have robust patient populations as well as strong research capabilities.

In this description of our study protocol, we describe the approach taken to conduct a multi-site prospective observational cohort study of PROs in our network of IM clinics. The Patients Receiving Integrative Medicine Interventions Effectiveness Registry (PRIMIER) provides an opportunity to better understand which IM interventions patients are receiving, and how these interventions impact both clinical measures and PROs, such as quality of life. Because randomized controlled trials can only examine one or two specific interventions for a narrowly defined condition—and because IM often uses highly individualized combinations of interventions to treat multiple conditions simultaneously—the evidence base to support clinical decision making in IM is not progressing rapidly enough to address the widespread use of this approach in our health care system [8]. There have been a few attempts to conduct randomized trials of IM interventions. However, these have been limited by: small sample size and inadequate power [9]; generalizability and resource intensiveness [10]; and retention challenges potentially related to participant dissatisfaction with being randomized to the non-IM arm, as well as limited follow-up [11].

## Methods

### Overview

In 2013, BraveNet created and launched PRIMIER (Patients Receiving Integrative Medicine Interventions Effectiveness Registry), a multi-institutional project designed to uniformly collect PROs and extract electronic health record (EHR) data into a large national registry. The goal of PRIMIER is to provide a framework that can be used for evidence-based practice, discernment of best practices of IM, and quality improvement. PRIMIER is listed in the Registry of Registries (RoPR ID: 40), which is maintained by AHRQ and ClinicalTrials.gov (NCT01754038). The intent is that PRIMIER will continue to expand over time, including more publicly-funded as well as private IM centers with ever increasing data which could be used for the mentioned objectives. At present, PRIMIER is 2 years into an anticipated 10-year study timeframe. Active enrolling sites are expected to grow significantly beyond the present 14 recruiting centers.

### Research setting and eligibility criteria

As of June 30, 2015, the BraveNet PBRN includes the 14 member sites listed in Table 1 as well as the BraveNet Data and Statistical Coordinating Center. Most of our sites are specialty care, integrative medicine clinics, although an increasing number of member clinics have primary care capacities. The study is open to any patient age 18 or over who is seen by a provider for clinical purposes at any of the sites. Study participants must be willing and able to provide informed consent, to participate, and to be contacted in the future by study personnel. Patients who are involved only in an education program or one-time activity are excluded from participation.

**Table 1:** BraveNet Member Clinics (as of 6/30/2015)

Site	Location
Alliance Institute for Integrative Medicine	Cincinnati, OH
Boston Medical Center at Boston University	Boston, MA
Center for Integrative Medicine at University of Maryland School of Medicine	Baltimore, MA
Duke Integrative Medicine at Duke University	Durham, NC
Integrative Medicine at the University of Colorado Denver	Denver, CO
Jefferson-Myrna Brind Center for Integrative Medicine at Thomas Jefferson Medical College	Philadelphia, PA
Mount Sinai Beth Israel Center for Health and Healing	New York, NY
Osher Center for Integrative Medicine at Northwestern University	Chicago, IL
Osher Center for Integrative Medicine at University of California, San Francisco	San Francisco, CA
Osher Center for Integrative Medicine at Vanderbilt University	Nashville, TN
Penny George Institute for Health and Healing at Allina Health	Minneapolis, MN
Scripps Center for Integrative Medicine at Scripps Health	La Jolla, CA
Simms/Mann Health and Wellness Center, Program in Integrative Medicine at Venice Family Clinic	Los Angeles, CA
University of Pittsburgh Center for Integrative Medicine	Pittsburgh, PA

### Ethics, consent and permissions

All participants will be asked to provide informed consent before initiation of any study-related procedures. The protocol was approved by the Institutional Review Board at each participating site including Beth Israel Medical Center Human Subjects Protection Office Institutional Review Board; Boston University Medical Center Institutional Review Board; Colorado Multiple Institutional Review Board; Duke University Health Systems Institutional Review Board; Northwestern University Institutional Review Board; Schulman Associates Institutional Review Board; Scripps Clinic Institutional Review Board; Thomas Jefferson University Division of Human Subjects Protection; University of California Los Angeles Institutional Review Board; University of California San Francisco Committee on Human Research; University of Maryland Institutional Review Board; University of Maryland Institutional Review Board and Vanderbilt University Human Research Protection Program. In addition, the Einstein Human Research Protection Program and Duke University Health Systems Institutional Review Board approved the study as the BraveNet Data and Statistical Coordinating Center. The study is registered in Clinical Trials.gov (NCT01754038).

### Screening procedures and enrollment

Potential participants receive PRIMIER enrollment information either from clinic or research staff. Patients who decide to participate in PRIMIER log onto the PRIMIER website and electronically enroll directly into the registry. Online registrants are able to provide informed consent upon opening the first screen on their initial visit to the website. If they do not have computer access or are uncomfortable with technology, patients may provide informed consent and complete the survey questionnaire by pen and paper. Following enrollment in PRIMIER, PRO measures are obtained at 2-month intervals for the first 6 months, then every 6 months through the end of year two. This information is combined with data extracted from the participants’ EHR. We anticipate approximately 10,000 participants to be enrolled in PRIMIER.

### Data collection

PRIMIER currently uses the Research Electronic Data Capture (REDCap) system as an online research management tool to collect patient reported data. REDCap is a secure, web-based application designed exclusively to support data capture for research studies [12]. REDCap, which was initiated with funding from the National Institutes of Health, allows researchers to create study-specific websites for capturing participant data securely through an intuitive interface for users to enter data and have real time validation rules (with automated data type and range checks) at the time of entry. REDCap further allows automated data export procedures for seamless data downloads to Excel, SPSS, SAS, and Stata. Developed by Vanderbilt University, REDCap currently supports 994 academic/non-profit consortium partners and over 100,000 research end-users (http://project-redcap.org). From August 2013 to May 2015, PRIMIER patient surveys were collected using the Patient Reported Outcomes Measurement Information System (PROMIS) Assessment Center at Northwestern University. The database was changed to REDCap in May 2015 to give the BraveNet coordinating center (Albert Einstein College of Medicine) the ability to modify the survey and create one registry database instead of maintaining individual site databases as had been necessary with the Assessment Center platform.

### Data collection schedule

With the exception of the tobacco and alcohol use questionnaires and EHR data, all other data measures are collected seven times: at baseline and at two, four, six, 12, 18, and 24 months. Tobacco and alcohol use are asked at baseline, 12, and 24 months, and medical record data are pulled at baseline and every 6 months. See Table 2 for the schedule of data collection, and the following Demographics, Outcome Measures, and Utilization/Intervention Data sections for more detail.

**Table 2:** Outcome Measures Utilized in PRIMIER

Measure	Frequency						
Demographics	Baseline	2 months	4 months	6 months	12 months	18 months	24 months
Age	●	●	●	●	●	●	●
Race	●	●	●	●	●	●	●
Ethnicity	●	●	●	●	●	●	●
Sex	●	●	●	●	●	●	●
Education	●	●	●	●	●	●	●
Marital status	●	●	●	●	●	●	●
Employment status	●	●	●	●	●	●	●
Insurance status	●	●	●	●	●	●	●
Likelihood of insurance billing	●	●	●	●	●	●	●
Household income	●	●	●	●	●	●	●
Self-reported height	●	●	●	●	●	●	●
Self-reported weight	●	●	●	●	●	●	●
Opioid medication use	●	●	●	●	●	●	●
Fruit and vegetable Intake	●	●	●	●	●	●	●
Tobacco and alcohol use	●				●		●
Exercise	●	●	●	●	●	●	●
Patient-Reported Outcome Measures							
PROMIS-29	●	●	●	●	●	●	●
PROMIS-1 (Quality of Life)	●	●	●	●	●	●	●
Perceived Stress Scale (PSS-4)	●	●	●	●	●	●	●
Patient Activation Measure (PAM)	●	●	●	●	●	●	●
Utilization/Intervention							
Patient Self-Report							
Experience with IM services	●	●	●	●	●	●	●
IM service utilization	●	●	●	●	●	●	●
Chronic pain	●	●	●	●	●	●	●
Primary condition being treated	●	●	●	●	●	●	●
Primary symptom being treated	●	●	●	●	●	●	●
Change in symptoms	●	●	●	●	●	●	●
Medical Records							
IM Clinic Visits							
Dates of all appointments to the IM clinic	●			●	●		●
IM services received	●			●	●		●
Diagnostic codes (ICD-9)	●			●	●		●
Billing codes (CPT)	●			●	●		●
Any Clinic Visit							
Height	●			●	●		●
Weight	●			●	●		●
Pain	●			●	●		●
Lipid Panels	●			●	●		●

### Demographics

PRIMIER participants are asked to answer 19 items relating to basic demographics: age, race, ethnicity, sex, education, marital, employment and insurance status, likelihood of insurance billing, household income, and self-reported height and weight. Lifestyle behaviors such as alcohol and tobacco use, nutrition and exercise habits, and use of opioid medication are also asked of all participants. These questions, shown in the Additional file 1, are not scored and will be treated categorically in data analyses.

### Patient-reported outcome measures

PRIMIER uses the PROMIS-29 instrument as the core PRO measure along with the Perceived Stress Scale-4 (PSS-4) [13] and the Patient Activation Measure (PAM) [14].

Created with funding from the National Institutes of Health, the PROMIS suite of PRO instruments provides clinicians and researchers access to efficient, valid, and responsive self-reported measures of health, including symptoms, function, and well-being. The PROMIS-29 instrument includes four-item short forms covering seven distinct domains including physical function, anxiety, depression, fatigue, pain intensity and pain interference, satisfaction with social role, as well as sleep disturbance. Questions are answered using standard one through five Likert scales. The PROMIS-29 has been validated as performing as well as a variety of legacy PRO measures [15], and the four-item subscales for depression and anxiety have been found to have good internal reliability among chronic pain patients [16]. A crosswalk has been developed and validated between PROMIS Pain Interference and the Pain Interference subscale of the Brief Pain Inventory [17], and between the PROMIS Fatigue short form and the Modified Fatigue Impact Scale [18]. PROMIS Depression has also been cross-walked with several common depression measures (e.g., Beck Depression Inventory-II and Center for Epidemiologic Study-Depression) [19]. In addition to the PROMIS-29, we include one PROMIS question to assess global quality of life.

The Perceived Stress Scale-4 (PSS-4) is a brief, validated, and widely used psychological instrument for assessing a participant’s perception of stress level. Based on an original 14-item scale [20], the PSS-4 consists of four questions to measure the degree to which participants perceive situations in their lives as stressful [13]. It includes questions related to perceived unpredictability, uncontrollability, and overload. This short version is recommended for a brief assessment when respondent time is limited. Participants choose responses ranging from never (0) to very often (4) with a total score ranging from 0 to 16. A recent study on the PSS-4 reconfirms its reliability [21].

The Patient Activation Measure (PAM) is a brief, validated instrument for gauging the knowledge, skills and confidence essential to managing one’s own health and healthcare [14]. The 13-item PAM assessment (a short form of the original 22-item instrument) [22] divides participants into one of four progressively higher activation levels. Each level addresses a broad array of self-care behaviors and offers insight into the characteristics that drive health activation [22]. Positive changes in activation have been associated with positive changes in a variety of self-management behaviors [23]. Improvement in patient activation has been shown to be strongly related to improvements in clinical outcomes such as decreased pain, increased utilization of prevention screenings, and a reduction in emergency room visits [23]. The four levels are defined as: Level 1 – Does not believe that he/she has an active or important role; Level 2 – Lacks confidence and knowledge to take action; Level 3 – Beginning to take action; and Level 4 – Maintaining behavior over time. The PAM has been used in several studies of IM interventions [24–26].

### Utilization/intervention data

In addition to providing PRO measures, participants report the primary condition(s) or symptom(s) for which they are receiving treatment and the type of IM practitioner from whom they are receiving care based on questions from the National Health Interview Survey. At each data collection point, patients are asked to rate or describe their change in symptoms, etc. since beginning care at the IM clinic [27]. Patients report whether they are experiencing chronic pain, defined as 4 or greater on an 11-point visual analog scale. PRIMIER also collects information on what IM interventions patients have utilized in the past 6 months. Utilization of IM treatments is captured through two methods: 1) as reported by the study participants using patient visit questionnaires; and 2) as reported by the sites through an electronic or paper medical record extraction process. Each site abstracts the following data points from their participant’s electronic medical and administrative records: dates of all appointments to the IM clinic, IM services received, diagnostic codes (International Classification of Diseases-9: ICD-9) associated with each IM visit, and billing codes (Current Procedural Terminology: CPT) associated with each visit. These data are encrypted and sent electronically to the BraveNet data coordinating center. Additional data fields will be added from patient visits to any clinic (i.e., not limited to IM clinic visits) as relevant to specific studies of interest, for example, height, weight, pain, and lipid panels.

### Data analyses

#### General statistical considerations

All subjects who complete baseline measurements, ≥1 follow-up visit at a BraveNet clinic, and who are followed for at least 6 months in the study will be included in statistical analyses. Counts and percentages will be reported on categorical variables whereas continuous variables will be expressed as means and standard deviations (SDs) and/or medians (25^th^ and 75^th^ percentiles). The Wilcoxon rank-sum test or Chi-square test (Fisher’s exact as appropriate) will be used to quantify comparisons as deemed necessary.

### Future data analysis

In observational studies like PRIMIER, there is no treatment randomization. Therefore, to minimize the effects of potential stable moderators (e.g., sex), channeling bias, and/or time- varying confounders, a Marginal Structural Model (MSM) approach will be utilized to analyze the final PRIMIER database. An MSM analysis is a weighted repeated measures approach using IM modality as a time-varying covariate as well as accounting for baseline characteristics. Weights produce a pseudo-population with a balance in both time-invariant and time-varying covariates, allowing for causal treatment comparisons using standard repeated measures models. The weighting will also be adjusted to account for missing data, providing validity under missing at random or missing completely at random. In order to incorporate adjustment for patients with missing visits, the same weight approach is used. However, instead of using a flag to designate IM modality, a flag denoting whether the patient remained in the study is used. The final weight for each patient’s observation is computed by multiplying the IM modality selection weights and the censoring weights. There are four major components which need to be computed/assessed to perform a MSM analysis: 1) weight estimates for each subject visit adjusting for IM modality, 2) weight estimates for each subject visit adjusting for study discontinuation, 3) an a priori chosen vector of time-independent variables, such as baseline characteristics, 4) an a priori chosen IM modality or set of IM modalities which will be assigned as the “treatment of interest” and tracked throughout time. Adjusted mean outcomes by study time as well as resulting F-test p-values will be reported by outcome measure.

As our sample size increases and the proportion of those completing long term follow-up assessments grows, we will be able to evaluate the impact of IM on pain scores in subgroups including but not limited to: sex, BMI < 30 vs BMI ≥ 30, and <40 on the PROMIS Depression Subscale vs ≥40 on PROMIS Depression Subscale. The potential of the varying impacts of IM on subgroups will be assessed by including interaction terms, i.e., subgroup by IM, in the MSM model. In the case that the interaction terms are statistically significant, separate MSM analyses will be performed within each subgroup. Also, the weight estimates for each subject visit will be computed adjusting for dose of modality or modalities of interest (e.g., acupuncture or IM physician visit) instead of IM modality in the MSM analysis. Accounting for modality dosing by visit over the course of the study may potentially create more robust weight estimates for each subject by providing more information than just a binary response variable.

Sensitivity analysis to assess the impact of retention rate will be performed on pre-specified subgroups of subjects who completed:

- 75 % of surveys over the study period
- 50 % of surveys over the study period
- 25 % of surveys over the study period

All analyses will be conducted using SAS (Cary, NC).

## Discussion

The BraveNet PBRN has the potential to provide valuable information regarding the benefits of IM in real world settings. The PRIMIER project is already demonstrating changes in PRO measures in participants receiving care at our collaborating clinical sites. At this early stage, the initial PRIMIER cohort contributing a full 6 months of data is not large enough to accurately assess which interventions or which combination of interventions have had the greatest effect on PAM scores, depression and stress scores, or other PROs. Nor is there yet the ability to discern which IM interventions are most effective at improving symptoms in specific clinical populations receiving care at the BraveNet clinics. However, as participation and the retention rate increase, we will be able to refine our analysis and further examine which modalities or combinations thereof are most effective. For example, we will be able to address the question of which IM approaches are most effective in treating pain, which is by far the most common reason that patients seek care at our clinics [28–30]. Moreover, what is the optimal dosing of acupuncture for specific cancer conditions or does elevated depressive symptomology influence the effectiveness of mind/body interventions in patients with physical ailments? As the PRIMIER database grows and with increased demographic diversity provided by additional future sites, BraveNet is well-poised to address some of the important questions that remain unanswered in the field.

The goal of PRIMIER is to create a registry of sufficient size so that we can explore subsequent hypotheses. For example, we may hypothesize that pain patients who receive acupuncture weekly for 8 weeks would have significantly improved outcomes on the PROMIS-29 measures at 12 months than pain patients who receive acupuncture less frequently than weekly. Another example of a hypothesis to be tested would compare the cohort of patients with baseline elevated depressive symptomology (<40 on the PROMIS Depression Subscale) vs non-depressive symptomology (>40 on PROMIS Depression) on Patient Activation Measure at 12 months.

From our prior mapping study of IM in the United States [31], and from a BraveNet Registry study [28–30], we learned that IM center leaders across the country perceived that their interventions were most useful in patients with complaints of chronic pain, depression, and stress. In a different study of patients with a chief complaint of chronic pain seen at the BraveNet sites, we previously reported that an IM approach to pain not only decreased pain, but depressive symptomology and stress as well [29]. Both of these studies were limited by virtue of either one-time only assessment [28–30] or small sample size [29]. From a more mature PRIMIER dataset, we should be able to ascertain which particular treatment modalities offer the greatest reduction in pain, as well as changes on measures of depressive symptomology and stress as examples.

The PRIMIER project offers a unique opportunity to assess the effectiveness of IM clinic intervention in a wide variety of clinical conditions. As the dataset grows and the duration of follow-up increases, we expect to be able to derive preliminary information to develop specific research proposals to address the difficult- to-treat conditions for which patients seek our care.

Given the rapid increase in the number of clinical settings offering IM services, the public is continuing to seek out these therapies to augment and integrate with conventional medical practices. While randomized controlled trials assess the efficacy of specific interventions for specific patient populations in “controlled settings”, observational studies evaluate the effectiveness of treatments in the real world of clinical practice. The highly individualized nature of IM interventions also can make the randomized controlled trials model problematic, since treatments often evolve over time as a function of a given individual’s response to treatment. Although there is now a growing body of randomized controlled trial research on the efficacy of integrative medicine approaches for a variety of conditions [32–38], in many ways this body of research does not accurately describe the real world practice of IM because of the controlled nature of the clinical trial paradigm. In contrast, observational research designs, despite some inherent limitations, present a promising option. Bell et al. suggest that observational designs are appropriate for studying complex interventions, despite the absence of randomization [2].

Treatment approaches consisting of multiple modalities or components present unique challenges. In their 2014 protocol of an IM primary care trial, Herman and colleagues provide a useful overview of how truly integrative approaches to care (as opposed to single-modality offerings) can be described as complex interventions [39, 40]. These complex interventions present challenges of “unpacking”, due to the wide range of potential treatment combinations and patient diagnoses [39]. Currently, we do not know what the makeup of the population for PRIMIER will be (i.e., what conditions and comorbidities patients will have and what combinations of therapies they might receive). For example, as in the Registry study [28], we may see a higher proportion of patients with pain as compared with other symptoms. Because our initial goal is to establish a registry from which to conduct subsequent research, a key feature of the project is that we are not driving the interventions or the population characteristics; rather, that makeup is clinically driven. Therefore, our work is subject to some limitations, from the standpoint of conventional health outcomes research.

Authors of other multimodal studies have cited limitations such as the inability to determine the relative efficacy of components in a multifaceted study or intervention, [11, 39] noting that the most beneficial elements of a intervention may vary from patient to patient depending on individual needs and personal characteristics [41]. Bell argues that treatments that combine modalities (conventional and/or complementary) should be *studied* in combination as well [2]. Herman notes that larger effect sizes may be achieved in more controlled settings [39]; however, this is a tradeoff for studying a heterogeneous patient population receiving individualized, sometimes complex care. Maiers et al. acknowledge the limited generalizability of an integrative care plan for low back pain, while also pointing out how the adaptability of integrative care models can make them more generalizable in some respects [10]. While data from individual PRIMIER sites may be similarly limited in their generalizability, the inclusion of sites and patient populations from around the country will provide a larger and more diverse sample than comparable studies to date.

Although we recognize the importance of including economic analyses in studies of IM, not all of the medical costs for enrolled patients’ care will be available to the extracts from our electronic health record (e.g., if a patient received care at an emergency department outside the study site health system). Every effort will be made to conduct appropriate health economic analyses with PRIMIER, but this is an important limitation. Any other limitations in our analyses will be discussed in future reporting of the study results. Notwithstanding, observational research using PROs will uniquely enable us to describe the results of this type of individualized and dynamic care [42].

The exploration of IM in real-world settings is consistent with the strategic plan of the National Institutes of Health, National Center for Complementary and Integrative Health (formerly the National Center for Complementary and Alternative Medicine). Specifically, by seeking to “increase understanding of ‘real world’ patterns and outcomes of [complementary and alternative medicine] use and its integration into health care and health promotion”, [43] PRIMIER will serve as a national platform for future evaluations of IM best practices. In providing the means to begin determining these best practices, the BraveNet PBRN will be able to define the most effective IM interventions in a timely fashion, making valuable information readily available that will help guide patients, providers, and payors to improve health and well-being for all.

## Additional file

Additional file 1:
**Lifestyle/Behavior Questions for PRIMIER.** (PDF 123 kb)

## Abbreviations

AHRQ: Agency for Healthcare Research and Quality
CPT: Current Procedural Terminology
EHR: electronic health record
ICD-9: International Classification of Diseases-9
IM: integrative medicine
MSM: Marginal Structural Model
PAM: Patient Activation Measure
PBRNs: practice-based research networks
PRIMIER: Patients Receiving Integrative Medicine Effectiveness Registry
PRO: patient-reported outcome
PROMIS: Patient Reported Outcomes Measurement Information System
PSS-4: Perceived Stress Scale-4 item version
REDCap: Research Electronic Data Capture
