# Supplementary material for: Patients Receiving Integrative Medicine Effectiveness Registry (PRIMIER) of the BraveNet practice-based research network: study protocol
Source: BMC Complement Altern Med. 2016 Feb 4;16:53. doi: 10.1186/s12906-016-1025-0 (PMC4743108; doi:10.1186/s12906-016-1025-0)
Supplement: Additional file 1: — Lifestyle/Behavior Questions for PRIMIER. (PDF 123 kb) [file 12906_2016_1025_MOESM1_ESM.pdf]

Appendix: Lifestyle/Behavior Questions for PRIMIER

<u>Tobacco and Alcohol Use</u> (Baseline, 12, 24 months)

13. Do you currently use tobacco products? *Examples of tobacco products are **cigarettes, cigars, pipes tobacco,** and **smokeless tobacco***	Yes ☐→→ (please continue to Q15)	No ☐ →→ (please continue to Q14)
14. Have you ever used tobacco products?	Yes ☐→→ (please continue to Q15)	No ☐ →→ (please continue to Q16)
15. How many years of tobacco use have you had?	__________________	
16. Do you currently drink alcohol? *Consider a drink to be a 12-oz can of beer, a 4-oz glass of wine, or one cocktail containing 1-oz of liquor*	Yes ☐ →→ (please continue to Q17)	No ☐→→ (please continue to Q19)
17. On average, how many days per week do you drink alcoholic beverages?	1☐ 2☐ 3☐ 4☐ 5☐ 6☐ 7☐	
18. When you drink alcoholic beverages, how many drinks, on average, do you have in a day?	__________________	

<u>Exercise</u> (Baseline 2, 4, 6, 12, 18, 24 months)
Please answer the following questions regarding your exercise habits.

19. Over the past <u>6 months</u>, on average, how often have you:

	**Never**	**Fewer than once per week**	**1-2 times per week**	**3-4 times per week**	**5 or more times per week**
Exercised aerobically for a minimum of 20 minutes? *(Examples of exercise are walking, running, and swimming for exercise)*	☐	☐	☐	☐	☐
Performed muscular strengthening exercises for a minimum of 20 minutes? *(Examples of muscular strengthening exercises are lifting weights, using therabands, and exercises like sit-ups and push-ups)*	☐	☐	☐	☐	☐

<u>Opioid Medication Use</u> (Baseline 2, 4, 6, 12, 18, 24 months)

32. Do you have a prescription from a health care provider for one or more of the opioid (narcotic) pain medications listed below? Check all that apply.

☐ Codeine (Tylenol 3®, Tylenol 4®)
☐ Fentanyl (Duragesic®)
☐ Hydrocodone (Vicodin®)
☐ Oxycodone (Percocet®,OxyContin®)
☐ Oxymorphone (Ophana®)
☐ Propoxyphene (Darvon®)
☐ Hydromorphone (Dilaudid®)
☐ Meperidine (Demerol®)
☐ Methadone
☐ Morphine (Kadian®, MS Contin®)
☐ I do not have a prescription for any of these medications (Skip Q33 and continue to Q34)

33. During the past week, on how many days did you take one or more doses of your opioid pain medications?

____

<u>Nutrition</u> (Baseline 2, 4, 6, 12, 18, 24 months)

34. How many servings of FRUIT do you usually eat or drink EACH DAY?
(Think of a serving as being about 1 medium piece, or ½ cup of fruit, or ¾ of cup of fruit juice)

0 1 2 3 4 5 6 7 8 9 10+

35. How many servings of VEGETABLES do you usually eat or drink EACH DAY?
(Think of a serving as being about 1 cup of raw leafy vegetables, ½ cup of other cooked or raw vegetables, or ¾ cup of vegetable juice)

0 1 2 3 4 5 6 7 8 9 10+
